# Is auditory discrimination mature by middle childhood? A study using time-frequency analysis of mismatch responses from 7 years to adulthood

**DOI:** 10.1111/j.1467-7687.2010.00990.x

**Published:** 2011-03

**Authors:** Dorothy VM Bishop, Mervyn J Hardiman, Johanna G Barry

**Affiliations:** Department of Experimental Psychology, University of OxfordUK

## Abstract

Behavioural and electrophysiological studies give differing impressions of when auditory discrimination is mature. Ability to discriminate frequency and speech contrasts reaches adult levels only around 12 years of age, yet an electrophysiological index of auditory discrimination, the mismatch negativity (MMN), is reported to be as large in children as in adults. Auditory ERPs were measured in 30 children (7 to 12 years), 23 teenagers (13 to 16 years) and 32 adults (35 to 56 years) in an oddball paradigm with tone or syllable stimuli. For each stimulus type, a standard stimulus (1000 Hz tone or syllable [ba]) occurred on 70% of trials, and one of two deviants (1030 or 1200 Hz tone, or syllables [da] or [bi]) equiprobably on the remaining trials. For the traditional MMN interval of 100–250 ms post-onset, size of mismatch responses increased with age, whereas the opposite trend was seen for an interval from 300 to 550 ms post-onset, corresponding to the late discriminative negativity (LDN). Time-frequency analysis of single trials revealed that the MMN resulted from phase-synchronization of oscillations in the theta (4–7 Hz) range, with greater synchronization in adults than children. Furthermore, the amount of synchronization was significantly correlated with frequency discrimination threshold. These results show that neurophysiological processes underlying auditory discrimination continue to develop through childhood and adolescence. Previous reports of adult-like MMN amplitudes in children may be artefactual results of using peak measurements when comparing groups that differ in variance.

## Introduction

It is difficult to study the development of the human auditory system using behavioural methods, because young children are less good than adults at concentrating on long repetitive sequences of sounds, and may lack the motivation and attention to comply with the psychophysical methods that are used to establish auditory thresholds. Performance on auditory tasks tends to improve from infancy to adulthood, but it can be hard to know how much of the improvement reflects changes in the physiology of the auditory system, and how much is due to improved ability to cope strategically with task demands, e.g. by identifying relevant features, focusing attention at the time point when an auditory stimulus is delivered, and sustaining attention over several minutes (Banai & Ahissar, 2006; Sutcliffe & Bishop, 2005; Werner & Marean, 1996). Animal models can provide information about development of the auditory system (Illing, 2004), and have demonstrated that auditory experience plays a critical role in normal development of auditory cortex, findings that have parallels in humans (D. Moore, 2002). Nevertheless, it is difficult to generalize across species, because different animals follow different maturational timetables. There are a few structural brain imaging studies of normal human development but they have not as yet focused specifically on the auditory system (Lenroot, Schmitt, Ordaz, Wallace, Neale, Lerch, Kendler, Evans & Giedd, 2009). There is intriguing evidence from small-scale neuroanatomical studies that development of auditory regions follows a protracted course during childhood (J. Moore & Guan, 2001; review: J. Moore & Linthicum, 2007), leading Eggermont and Ponton (2003) to suggest that maturation of axons in layer II and upper layer III of the auditory cortex might account for developmental improvements in auditory skills such as speech perception in noise, which continues to improve throughout childhood. Nevertheless, direct evidence for correlations between behaviour and physiology is lacking.

Electrophysiological methods, and their magnetic counterpart, magnetoencephalography, are currently the most promising approach to study functional auditory development in humans. The most widely used method for investigating auditory discrimination is the mismatch negativity (MMN), which involves comparing brain waves elicited by a repeated standard sound with those elicited by a rarer deviant sound differing on some acoustic dimension (Näätänen, Paavilainen, Rinne & Alho, 2007). The MMN is typically measured in situations where the participant is not required to attend to or respond to the sounds, and so can be regarded as reflecting automatic detection of the change in sound between standards and deviants. It constitutes an enhanced negativity occurring around 100–250 ms post-stimulus onset.

There has been considerable interest in the MMN as a measure of auditory system development (Cheour, Leppänen & Kraus, 2000). For children aged 6 years and over, it has been argued that the MMN has a longer latency than in adults, but its amplitude is as large, if not larger, than the adult MMN (Cheour, Korpilahti, Martynova & Lang, 2001). Furthermore, children show a later negativity, the ‘late MMN’ or late discriminative negativity (LDN), around 300–550 ms after the onset of the stimulus difference. The LDN is most prominent in response to speech and was originally thought to reflect lexical processing (Korpilahti, Krause, Holopainen & Lang, 2001), but it can also be elicited by non-speech sounds. Although it can be seen in adults, it is particularly pronounced in children (Cheour *et al*., 2001; Kraus, McGee, Carrell, Sharma, Micco & Nicol, 1993). Taken together, these findings would seem to indicate that the brain’s ability to discriminate sounds is well developed in childhood, and that poor behavioural discrimination is due to other factors.

Nevertheless, it is debatable how far the mismatch responses described in children resemble those seen in adults, in terms of their underlying causes, reliability, topography and time-course (Picton & Taylor, 2007). Also, there are exceptions to the reports of larger MMNs in children than adults: for instance, Oades, Dittmann-Balcar and Zerbin (1997) found larger MMN to tones in young adults than in 10- to 14-year-olds. A potential reason for disagreement has to do with how MMNs are measured. This is typically done by first computing separate averages to standard and deviant stimuli across many trials, subtracting these to give a difference wave, and then taking the mean or peak amplitude of the difference wave in a given time window. Peak measures have been used in most developmental studies, but they are problematic because ERPs typically have greater variance in children than adults. If we have two waveforms with the same average amplitude, but one is much noisier than the other, then a measure of mean amplitude over an interval will be similar in both cases. However, a directional measure, i.e. a minimum corresponding to a negative peak, will be larger for the noisier waveform. This is a statistical inevitability because deviations from the mean will be larger in both directions. It is noteworthy that the study by Oades *et al*. (1997) would not have been subject to this artefact because amplitudes were normalized, so any age differences in variance were eliminated.

**Figure 1:**
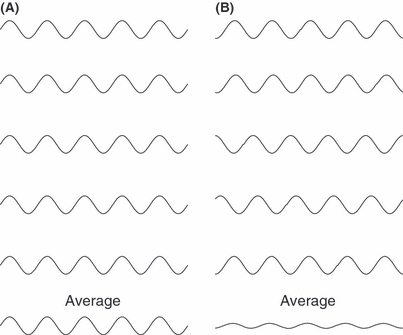
Illustration of phase-synchronization account of ERP waveforms. Panel A shows five sine waves in phase, which give an averaged waveform of the same form and amplitude; Panel B shows five sine waves of identical amplitude with random phase; when these are averaged, they will tend to cancel out to give a flatter waveform.

Reliance on measures of peak amplitude calculated from an average waveform has a further problem: information about underlying oscillatory mechanisms that are involved in generation of auditory ERPs is lost. The traditional rationale for averaging of ERPs is that it allows one to detect neural responses occurring in a background of noise, with the implicit assumption that the response of interest is a neuronal activation consisting of an increase in amplitude of a given polarity during a specific time window. However, it has been pointed out that background EEG activity is not random, but rather consists of an ensemble of oscillations at different frequencies (Başar, Başar-Eroglu, Parnefjord, Rahn & Schürmann, 1992). Sayers, Beagley and Henshall (1974) were the first to note that there was no difference in spectral power between the auditory potential measured after an audible tone vs. an inaudible tone, although the averaged ERP was markedly different in the two cases. They concluded that the characteristic peaks and troughs in the auditory ERP to a detectable tone were caused by synchronization of the phase of the spectral components present in the spontaneous activity (see Figure 1). On this view, the peaks seen in the averaged waveform result from temporal synchronization of existing neural activity rather than recruitment of newly activated neurons. This phase-resetting account of ERPs has subsequently been extended to account for a wide range of EEG phenomena (Başar, Başar-Eroglu, Karakas & Schürmann, 1999). Frequency-domain (spectral) analysis has been applied to auditory ERPs (e.g. Fuentemilla, Marco-Pallarés & Grau, 2006; Mülller, Gruber, Klimesch & Lindenberger, 2009; Schürmann & Başar, 1994), but its application to the MMN is still in its infancy. Open-source EEGLAB software of Delorme and Makeig (2004) simplifies the calculation of two complementary indices in the time and frequency domains from single-trial data (Makeig, 1993; Makeig, Debener, Onton & Delorme, 2004). The first, event-related spectral perturbation (ERSP), measures the extent to which there is increase or decrease in power at a given frequency range associated with an event onset, measured relative to pre-stimulus baseline. It expresses a ratio and is usually measured in dB. In Figure 1, the ERSP would be equivalent in panels A and B, with a high value if the oscillatory waveform had been preceded by a non-oscillatory baseline, and a low value if the same oscillations were present in the baseline. The second index, inter-trial coherence (ITC), measures the extent to which activity at a given frequency is in phase across different trials. It is a unitless measure that takes values from zero to one – e.g. for Figure 1, panel A would show perfect ITC of one, and panel B would show zero ITC. If ITC is higher after stimulus onset than during baseline, this indicates event-related phase-locking.

There have been three studies investigating temporal synchronization of the MMN. Hsiao, Wu, Ho and Lin (2009) compared phase-locking of event-related fields to standard and deviant (shorter duration) tones using magnetoencephalography, and found an increase of phase-locking specific to the deviant tones at 150–200 ms after stimulus onset only in the theta frequency range (4–8 Hz). Their findings were in broad agreement with Fuentemilla, Marco-Pallarés, Münte and Grau (2008), who found greater ITC in the theta frequency band for deviant than for standard stimuli in an auditory oddball design. ERSP was higher for deviants than standards at frontal but not temporal sites. Bishop and Hardiman (2010) adopted a rather different approach, performing single trial analysis of difference waveforms created by subtracting deviant trials from the preceding standard. They found no evidence for event-related spectral power changes in these waveforms, but there was significant ITC at the theta frequency, indicative of event-related phase resetting.

With regard to children, maturational changes in the spectral composition of the resting EEG have been reviewed by Yordanova and Kolev (2008). As children mature, there is a relative decrease of power in the theta range (4–7 Hz), with a corresponding increase in alpha (7–13 Hz). During adolescence, there is a continued decrease in spectral power below 8 Hz, which has been linked to loss of gray matter during synaptic pruning (Whitford, Rennie, Grieve, Clark, Gordon & Williams, 2007). Despite these changes in background spectral power, auditory ERPs are characterized by a burst of power in the theta frequency range in children as well as adults (Kolev, Rosso & Yordanova, 2001). Furthermore, single-trial analysis of auditory ERPs revealed that theta phase-locking increased with age during childhood and adolescence (Mülller *et al*., 2009; Yordanova & Kolev, 2008; Shahin, Trainor, Roberts, Backer & Miller, 2010). Since theta phase-locking increases with age and is larger for deviant than for standard stimuli (Fuentemilla *et al*., 2008; Hsaio *et al*., 2009; Mülller *et al*., 2009), we might predict that the MMN should develop with age. This prediction does not, however, seem to be supported by the literature to date, where MMN development has been studied using time-domain analyses. It is therefore of considerable interest to ask whether time-frequency analysis will shed new light on development of mismatch responses.

In the current study, we compared mismatch responses in children, adolescents and adults using a paradigm based on Uwer, Albrecht and von Suchodoletz (2002), in which a standard stimulus (either a pure tone or a CV syllable) was presented together with two rarer deviants (tones of different frequencies, or syllables with different phonemes). We assessed changes with age for mismatch responses in both the classic MMN time window and in the LDN window, considering spectral composition and phase-locking features, as well as traditional measures of peak amplitude. In particular, we considered the questions of (a) Does the MMN change with age? (b) Do children differ from adults in the extent to which their mismatch responses can be accounted for in terms of phase-locking of evoked responses? (c) Are developmental changes in mismatch responses related to improvements in behavioural measures of frequency discrimination? In addition, we considered the supplementary question of whether the LDN differed from the MMN, either in terms of age trends or in terms of sensitivity to stimulus type.

## Methods

### Participants

Participants were typically developing children, recruited from Oxfordshire state schools, and their parents. Families where the child had evidence of language or literacy problems were excluded from consideration. On the basis of prior research by Bishop, Hardiman, Uwer and von Suchodoletz (2007), participants were grouped into three age bands: 7 to 12 years, 13 to 16 years and adults. Six potential participants were excluded because they had fewer than 80 epochs per deviant after artefact rejection (see below), giving final group sizes of 30 children (7 to 12 years), 23 teenagers (13 to 16 years) and 32 adults. Nonverbal IQ was assessed using the Wechsler Abbreviated Scale of Intelligence (Wechsler, 1999) performance subtests. Time constraints sometimes meant further psychometric testing was not possible, but the Test of Word Reading Efficiency (Torgesen, Wagner & Rashotte, 1999) and the Test for Reception of Grammar (Bishop, 2003) were administered to 25 children aged 7 to 12 years, 19 of those aged 13 to 16 years, and all the adults, and confirmed that the mean and range of scores on language and literacy skills were in line with the general population (see Table 1). It was not possible to achieve a gender balance in the sample, because males and females were not equally likely to volunteer: in particular, mothers were more often available for testing than fathers. The study was approved by the Oxford Psychiatric Research Ethics Committee; parents of all participants gave written informed consent, and the children themselves gave assent after the study was explained in age-appropriate language.

**Table 1:** Mean ( SD) age, age-scaled test scores, and numbers of accepted deviant epochs for participants by age group

	7 to 12 yr 18 f, 12 m	13 to 16 yr 8 f, 15 m	Adult 26 f, 6 m
Age (yr)	10.1 (1.37)	13.9 (0.95)	43.7 (5.33)
Nonverbal IQ	109.1 (15.36)	104.0 (16.46)	113.6 (11.44)
TOWRE word	102.5 (12.38)	97.6 (11.96)	93.0 (12.51)
TOWRE nonword	108.2 (12.75)	105.7 (11.26)	99.1 (12.58)
TROG	101.2 (10.79)	104.1 (5.29)	101.7 (7.87)
*N* deviant epochs: tones	87.9 (12.80)	92.3 (11.35)	97.9 (12.85)
*N* deviant epochs: syllables	86.9 (14.79)	92.5 (9.45)	99.1 (10.46)

### Psychoacoustic assessment of frequency discrimination

Discrimination thresholds were measured for tones only, as speech sounds are perceived more categorically. Stimuli were presented on a laptop computer using Sennheiser HD25 headphones. A self-paced AXB three-alternative two-interval format was adopted, using software developed for prior studies (Sutcliffe & Bishop, 2005). The participant heard three tones on each trial, and was asked to select the tone (first or third) that differed from the middle one. A cartoon dinosaur was shown on the screen to correspond to each sound. All tones were presented at 85 dB SPL. The standard stimulus, corresponding to the middle dinosaur, was a 1000 Hz tone of 100 ms duration. The first or third tone was either another standard stimulus or a deviant stimulus of higher frequency. The initial frequency difference was set at 1200 Hz, which is usually found to be clearly discriminable, and the frequency was adjusted adaptively depending on the participant’s responses using a ‘more virulent PEST’ procedure (Findlay, 1978). This reduces the difference between the standard and deviant tones progressively after correct responses, and increases it after errors, initially using large steps and then moving to smaller steps to converge on a threshold corresponding to 75% correct. Correct responses were rewarded by adding to a set of icons that were presented on the screen, accompanied by a cheerful noise. After errors, no icon was added, and a ‘sigh’ sound was presented. The main test run was preceded by practice trials without headphones, during which the experimenter demonstrated the correct response to the participant, and gave verbal feedback and encouragement until it was clear that the task had been understood. In general, two runs were given, unless a low threshold (10 Hz or less) was achieved on the first run. Thresholds (in Hz difference from the standard) were converted to natural logs to normalize the data. Because inattention can produce spuriously high psychoacoustic thresholds, especially in children (Sutcliffe, Bishop, Houghton & Taylor, 2006), the better of the two estimates was treated as the best estimate of true threshold, and was used in the analysis.

### Electrophysiological procedure

#### Stimuli

Tone stimuli consisted of a standard 1000 Hz sinusoid and deviants of 1200 Hz (large deviant) and 1030 Hz (small deviant). Speech stimuli consisted of the syllables [ba] (standard), [bi] (large deviant) and [da] (small deviant). Consonant burst time differences were minimized for the natural speech stimuli, the intonation contours were equated using Praat (Boersma & Weenink, 2005) and the final stimuli were RMS equalized with GoldWave (Craig, 2008). Thus consonant change detection was primarily based on formant transitions into the vowel. We defined degree of deviation based on time available for change detection, i.e. the formant transitions into steady state portion of the vowel for [da] occurred over a period of 68 ms from voice onset which was relatively short compared to the duration of the steady state vowel duration (141 ms) of the deviant [bi]. All stimuli had durations of 175 ms, windowed at 15 ms, and were presented monaurally to the right ear at 86.5 dB SPL through sound attenuating Sennheiser HD25-1 headphones.

#### Procedure

Standards were presented on 70% of trials, with each deviant occurring on 15% of trials in a quasi-random sequence, avoiding occurrence of two deviants in succession. Stimulus onset asynchrony was 1 s. There were two blocks each of 333 trials, making a total of 466 standards, and 100 of each deviant type. In some cases, additional trials were run if the session had included a large proportion of noisy trials.

Participants were seated in a comfortable upright chair in a sound-attenuated electrically shielded booth. Where feasible, a parent and child were tested simultaneously, seated side by side. To help them ignore the stimuli, participants played Gameboy, watched a DVD, or a self-selected silent video on a small television located at a distance of 1.3 m away.

#### EEG recording and data analysis

The EEG was recorded on a SynAmps or NuAmps NeuroScan Inc. system using Ag/AgCl sintered electrodes and a water-soluble conductive gel. Early pilot studies indicated no difference in the results obtained from the two recording systems.

An electrode cap was fitted to record from 28 sites: FC1, F7, FP1, FZ, FP2, F8, FC2, FT9, FC5, F3, FCZ, F4, FC6, FT10, T7, C3, CZ, C4, T8, CP5, P7, P3, PZ, P4, P8, CP6, M1, and M2. M1 or M2 was selected as reference electrode and ground was placed at AFZ. Electro-oculograms (EOG) were recorded from supra- and infra-orbital electrodes on the left eye and also from electrodes placed lateral to the left and right eyes. Impedances for all electrodes were kept below 8 kΩ. The EEG was recorded continuously on-line and stored for off-line processing. EEG data were digitized at 500 Hz and band-pass filtered (0.01–70 Hz for SynAmps; 0.1–70 Hz for NuAmps) and a 50 Hz notch filter was employed.

#### Data processing

##### Overview

The steps of data processing included (a) pre-processing and artefact rejection of trials with extreme amplitudes; (b) artefact reduction, using independent component analysis (ICA) to identify unwanted components, which were mathematically subtracted from the data; (c) spatial principal components analysis (PCA) to create a single channel for analysis using weights of the largest principal component; (d) subtraction of the average standard response from all trials, to give three types of difference waves: one for each deviant, and one ‘dummy’ set based on standards; (e) conventional analysis of peak measurements for MMN and LDN for each type of difference wave; (f) single-trial analysis to measure ERSP and ITC for each type of difference wave. Details of each step in the analysis will now be presented.

##### a. Artefact rejection of extreme trials

Raw EEG data were downsampled to 250 Hz to facilitate efficient data processing, high pass filtered at 0.5 Hz to remove drift, re-referenced to the mean mastoids to remove any lateral bias, and divided into 1000 ms trials including a baseline of 200 ms. The data were then baseline corrected, and trials with amplitude great than ± 300 μv were removed. Use of this high cutoff ensured we removed noisy sections of the record but retained trials with eyeblinks.

##### b. Artefact reduction

Eyeblinks and other focal artefacts were detected using second-order blind identification (SOBI) independent component analysis (Tang, Sutherland & McKinney, 2005), implemented in EEGLAB software (Delorme & Makeig, 2004). Like other methods of independent component analysis, SOBI decomposes the EEG into a mixture of independent sources, which generally correspond to physiologically plausible generators, but unlike more traditional methods, it is sensitive to temporal as well as spatial features of the data. We found that an objective algorithm applied to the output of SOBI was effective in identifying sources due to blinks and horizontal eye-movements. This involved standardizing the inverse weight matrix that contained the weightings of each channel in relation to each component, and then selecting components that included an extreme weight (absolute *z*-score of 4 or more). Components meeting this criterion were then mathematically removed from the data by back-projection, effectively removing artefacts without needing to delete the trial.

##### c. Spatial principal component extraction

A PCA was done participant-by-participant, treating channel amplitudes at each time point in the averaged waveform (all trials) as a new set of observations. Weights from the first component were then used to create a new channel consisting of the weighted average of all channels. Because the polarity of the weightings from PCA is random (e.g. a large contribution from Fz could be indexed by either a large positive or large negative weight), the average signal for the new PC channel was correlated with the average from the Fz channel, and its polarity was reversed if the correlation was negative. The weights for participants in each group were averaged to provide an overall measure of topography of the PC channel for that group. In addition, the percentage of variance accounted for by the first component was recorded for each participant.

##### d. Computation of difference waves

Trials were sorted into deviants (two types per condition), standards preceding a deviant, and other standards. Because we aimed to study single trials as well as overall averages, for each participant the grand average for standards (other than those preceding a deviant) was subtracted from each trial, to give a set of mismatch responses (see Bishop & Hardiman, 2010, for an analogous approach where the preceding standard was subtracted from each deviant trial). This gave a set of epochs for each deviant, each consisting of a difference wave. As recommended by Picton and Taylor (2007), we also created ‘dummy’ difference waves based on standards that immediately preceded deviants. By comparing mismatches seen on these ‘dummy’ trials, where standards and dummy deviants were identical, with genuine mismatch trials we could estimate the validity and reliability of mismatch responses. The mismatch responses were then low-pass filtered using an IIR filter with cutoff of 30 Hz to smooth the peaks, and trials with absolute amplitudes greater than 100 μv were removed before baseline correction was reapplied. Finally, spatial PCA was re-run on the average difference wave for each deviant type, and the spectral power of the first component was computed for the frequency range 5 to 20 Hz (i.e. including theta and alpha frequencies) using the spectopo function from EEGLAB software. This conducts a fast Fourier transform across the whole epoch, i.e. it estimates power in different frequency bands, but is not sensitive to time.

##### e. Conventional analysis of peaks

Mean difference waves were computed for each kind of mismatch (dummy, large deviant and small deviant) for tones and syllables. Significance of mismatch responses at the group level was tested using *t*-tests at each time point; this analysis was conducted separately for the dummy and both types of mismatch wave, in each case comparing the collection of average amplitudes in a group with zero. As has been noted by Guthrie and Buchwald (1991), significance levels of *t*-values are misleading with this approach, because time points are not independent of each another. However, the likelihood of obtaining spurious differences can be directly estimated by considering the dummy difference waves, where the same stimulus acts as standard and deviant. Mean amplitude of the difference wave was measured both in the interval corresponding to the MMN (100–250 ms) and the LDN (300–550 ms).

##### f. Single-trial time-frequency analysis

EEGLAB software was next used to run time-frequency analysis, using the newtimef function, to obtain measures of ERSP and ITC. In this analysis, spectral power is measured at different points in time during an epoch, using wavelet analysis. Note, however, that temporal resolution is poor, especially at low frequencies. The frequency range was specified as 4–16 Hz. The default settings were used, with pad ratio of four; this gives ITC and ERSP for seven equally spaced frequency bins from 3.9 to 15.6 Hz; the first three of these were averaged to estimate theta values. To assess significance of these indices at the group level, we subtracted the mean index (ERSP or ITC) for the dummy difference wave at each time point from the same index based on true mismatch waves, and then conducted a *t*-test across participants in a group. In effect, this method treats the dummy trials as providing an indication of the size of effect to be expected if there is no difference between standards and deviants.

## Results

### Topography of auditory ERPs at different ages

**Figure 2:**
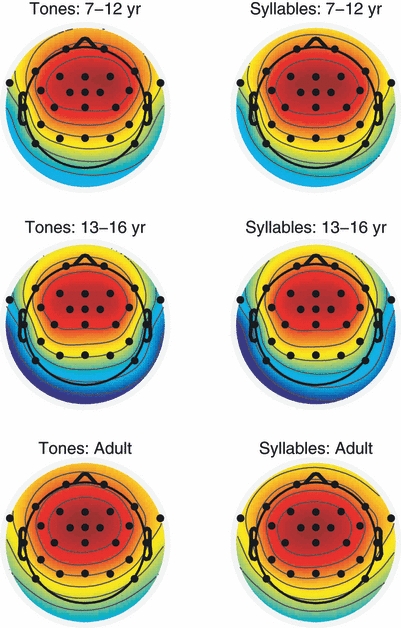
Topographic plots showing averaged weightings of electrodes on first principal component, in relation to age and stimulus type; arbitrary scaling from negative (blue) to positive (red). The average percentage variance accounted for by the principal component is shown beneath each plot.

In basing the analysis on a spatial principal component, we ensure that for each participant we include those electrodes contributing most to the auditory ERP. It is thus of interest to know whether a different topography is represented at different ages. Figure 2 shows the topography of the first principal components for the three age groups and the two stimulus types for the auditory ERP (all stimuli, prior to computation of the difference wave). It is evident from inspection that the topography is closely similar in all groups, with the fronto-central distribution that has been characteristically described for auditory ERPs.

### Mean amplitude of auditory ERPs at different ages

**Figure 3:**
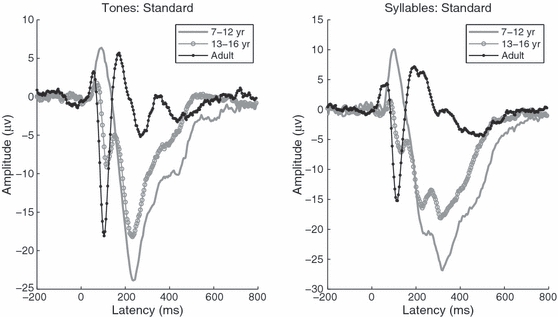
Mean amplitude of response to standards for principal component in relation to age and stimulus type.

Figure 3 shows mean amplitudes of the auditory ERP to standard stimuli for the three age groups and two stimulus types. The pattern is consistent with that previously reported by Bishop *et al*. (2007) with a similar paradigm. The N1 is not seen in the youngest age group, 7- to 12-year-olds, who instead show a large P1 around 100 ms followed by a large N2 around 200–300 ms. For the 13- to 16-year-olds, the N1 is clear, especially for tone stimuli, but still much smaller and later than in the adults, who also show a large P2 around 200 ms, which is absent or minimal in the younger groups.

### Mean amplitude of mismatch responses

**Figure 4:**
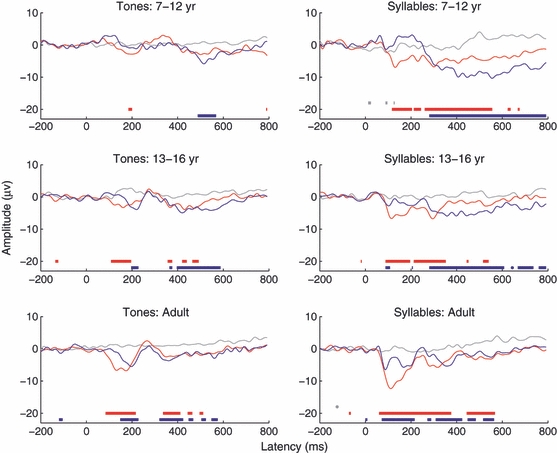
Mean amplitude of difference waves, after subtracting average standard wave, for dummy mismatch (grey lines), large deviants (red lines) and small deviants (blue lines). The same colour coding is used at the bottom of each plot to depict regions where a *t*-test gave a value below −1.96 when comparing the average amplitude with zero.

The average number of deviant epochs for each group is shown in Table 1. The mismatch responses for both kinds of deviant and for dummy deviants are shown in Figure 4, together with bars denoting regions where the *t*-value falls below −1.96. For tones, significant regions of mismatch are found for both large and small deviants in the traditional interval for the MMN, 100–250 ms, for the teenagers and adults, but the children show only a brief period of significance for the larger deviant. All groups show a more prolonged period of negativity in the LDN interval between 300 and 600 ms post-onset, but for children this is significant only for the small deviant. With syllables, significant mismatch in the interval 100–250 ms is clear for the large deviant in all groups, but for the small deviant it appears to show a developmental trend, being absent in the youngest group, very brief for the 13–16-year-olds, and clear in adults. For both stimulus types, the LDN is more pronounced for the small mismatch than for the large difference in children. In adults, although there is a significant negativity in the late interval, it is less prolonged than for children, and is of similar size for small and large deviants. Note that, despite the interdependence of the time points for which *t*-values were computed, we can be confident that these are not spurious results because regions of significant mismatch were rare using this criterion when the dummy difference waves were used (shown as grey bars in Figure 4).

**Figure 5:**
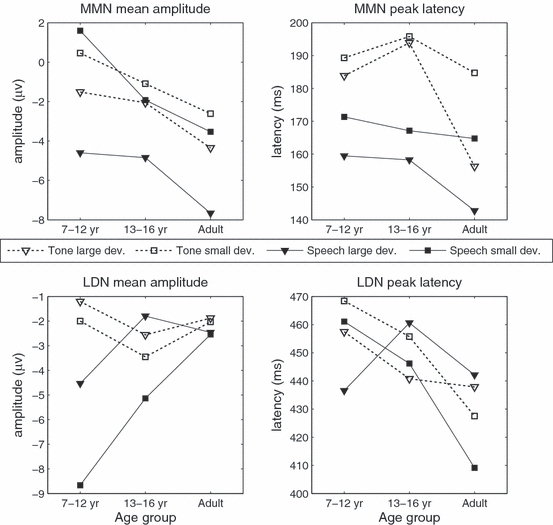
Mean amplitude of difference wave in (upper panel) 100–250 ms window, and (lower panel) 350–550 ms window, from dummy mismatch and true mismatch waves for tone and syllable stimuli. Deviant 1 is large deviant and deviant 2 is small deviant.

To test the significance of age effects on mismatch responses, analyses were conducted on mean amplitudes of the principal component measured in the traditional MMN window (100–250 ms) and in the later time window where an LDN could be seen (350–550 ms). Analysis of variance was conducted, with factors stimulus type (tone or syllable) and deviant type (large or small) as repeated measures, and age group (1, 2 or 3) as between-subjects factor, with a polynomial contrast. This allowed us to assess linear changes in mismatch response with age group, as well as interactions between age group and stimulus characteristics; main effects of stimulus type were of less interest because of the arbitrary nature of the stimuli. A significance level of *p* = .025 was used to take into account the likelihood of spurious findings when conducting one ANOVA for each of two intervals. Effect sizes are reported as partial eta square (η^2^), and full ANOVA outputs are reported in Supplementary material. For the early interval from 100 to 250 ms, there was a significant effect of age group, *F*(2, 82) = 9.86, *p* = < .001, η^2^ = .19, but no interactions with age. The main effect of stimulus type fell short of significance, but there was a large effect of deviant type, *F*(1, 82) = 26.2, *p* < .001, η^2^ = .24. Contrast analysis indicated a significant linear trend for age group, *p* = .001. As can be seen in Figure 5, the age effect reflected the increasing size of mismatch response with age group. For the later interval from 350 to 550 ms, there was a significant effect of deviant type, *F*(1, 82) = 7.9, *p* = .01, η^2^ = .09, but the effect went in the opposite direction to that seen for the MMN, with more negative LDN for small than for large deviants. The effect of age group was not significant, *F*(2, 82) = 1.76, *p* = .18, with linear trend *p* = .065. There was a marginal trend for an interaction between stimulus type and age group, *F*(2, 82) = 3.6, *p* = .03, η^2^ = .08. This was explored further with an analysis restricted to syllable stimuli. This now showed a significant effect of age group, *F*(2, 82) = 9.3, *p* < .001, η^2^ = .19, with a strong linear trend, *p* < .001. Note, however, that the age effect was in the opposite direction to that seen for the MMN, with larger mean amplitudes in the youngest group.

Similar ANOVAs were conducted on peak latencies. For the early MMN interval, there was a significant effect of age group on latency, *F*(2, 82) =3.98, *p* = .02, η^2^ = .09, with a marginal linear trend indicating a reduction of latency with age, *p* = .026. The main effects of deviant type and stimulus type were also significant, deviant type: *F*(1, 82) = 9.06, *p* < .001, η^2^ = .10; stimulus type: *F*(1, 82) = 16.1, *p* < .001, η^2^ = .16. The larger deviant gave an early MMN, and MMNs to speech were earlier than those to tones. For the later LDN interval, there was also a significant effect of age group, *F*(2, 82) =5.06, *p* = .01, η^2^ = .11, with a significant linear trend, *p* = .003, indicating earlier mismatch responses in the older groups. No other main effects or interactions were significant.

### Spectral power of mismatch waves

**Figure 6:**
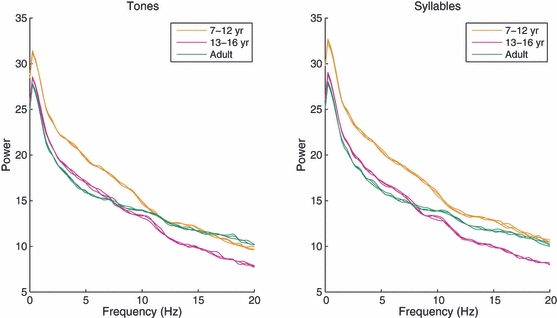
Power (dB) relative to frequency (Hz) for difference waves in relation to age. The plot shows three lines for each age corresponding to dummy waves and two kinds of mismatch wave, but these are not differentiated by symbols, as they are virtually superimposed.

Figure 6 shows the mean spectral power for the mismatch waves in each age group. The plots for dummy waves and the two kinds of mismatch wave are so similar that they cannot be distinguished within an age group, indicating that genuine mismatch is not associated with any increase in spectral power, even though the amplitude of the genuine mismatch average response is much greater than that of the dummy mismatch. The most striking features of Figure 6 are (a) the trend for overall power to decline with age for frequencies below 7 Hz, and (b) the divergence of the plots for 13- to 16-year-olds and adults in the region above 8 Hz, indicative of a boost in power in the alpha range for adults. Note, however, that this characterizes responses to dummy mismatch stimuli as well as to genuine mismatch.

### Inter-trial coherence and event-related spectral perturbation of mismatch waves

**Figure 7:**
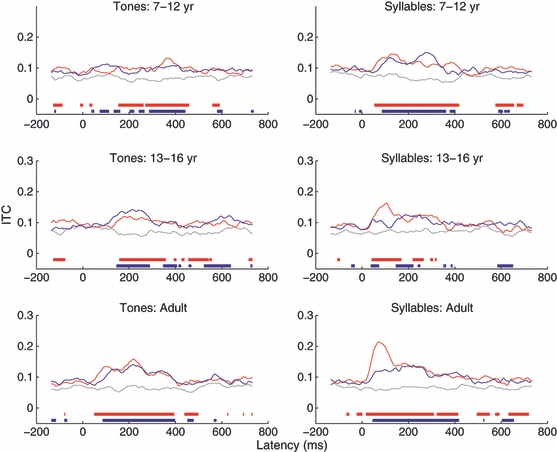
ITC in the theta range by time for difference waves in relation to age group and deviant type.

**Figure 8:**
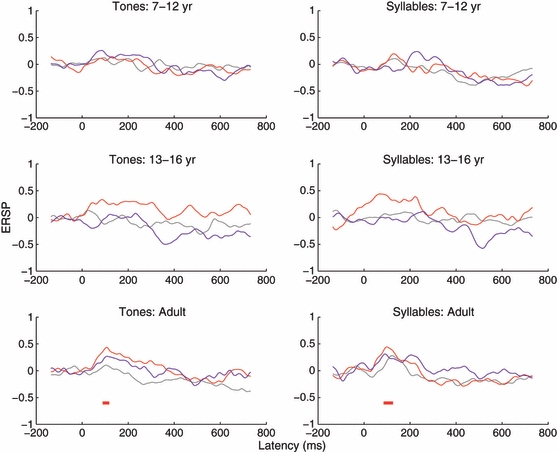
ERSP in the theta range by time for difference waves in relation to age group and deviant type.

If there are differences in amplitude between dummy and true mismatch waves but no differences in spectral power, this suggests that the mechanism underlying the amplitude difference must be related to phase coherence. Figure 7 indicates that this is indeed the case. The ITC values for the two mismatch stimuli lie above those for the dummy mismatch in all conditions at all ages, peaking around the interval when the MMN is seen. In contrast, the only cases where ERSP of true deviants was for a brief period around 100 ms in adults for large deviants (Figure 8).

Statistical analysis was conducted in a similar fashion to the mean amplitude analysis, with repeated measures of stimulus type (tone or syllable) and deviant type (large or small), and age group (1, 2 or 3) as between-subjects factor. Temporal resolution of time-frequency analysis is relatively poor, so a broad division was made of the epoch into an early portion (0–300 ms post-onset) and a later portion (300–600 ms post-onset), with mean ITC or ERSP computed for each individual. For ITC in the early interval, there was a significant effect of age, *F*(2, 82) = 7.35, *p* < .001, η^2^ = .15, with a significant linear trend, *p* < .001, indicating that mean values increased with age. The effect of deviant type fell short of significance. There was no age effect on ITC in the later interval. The effect of age also fell short of significance for ERSP in both early and late intervals, though in both cases there was a significant interaction between age and deviant; early interval: *F*(2, 82) = 5.18, *p* = .01, η^2^ = .11; late interval: *F*(2, 82) = 5.87, *p* < .001, η^2^ = .13. This is hard to interpret and could be a chance finding: it appears to reflect a greater separation between small and large deviants for the teenagers (see Figure 8).

### Correlations between behavioural and physiological responses to tones

Data on frequency discrimination were missing for four children aged 7 to 12 years and four teenagers, either because of unwillingness to undertake further testing after a lengthy ERP session, or because of equipment failure. For the remaining participants, the mean log frequency difference for behavioural thresholds was 3.10 (*SD* = 1.31) for 7- to 12-year-olds, 2.1 (*SD* = 1.07) for 13- to 16-year-olds, and 2.03 (*SD* = 1.48) for adults. One-way ANOVA confirmed that there was a significant effect of age, *F*(2, 74)= 5.6, *p* = .005, partial η^2^ = 012, and post-hoc Scheffé tests indicated that the youngest group differed significantly at the .05 level from the two older groups, who did not differ from one another. In original units, the mean frequency difference thresholds for the three groups translate into 22.2 Hz, 8.23 Hz and 7.61 Hz; these values are consistent with control data from other studies on younger children and adults using a 1000 Hz standard in a similar paradigm (6–13-year-olds, 21.7 Hz; Halliday & Bishop, 2005; adults, 6.7 Hz; Heath, Bishop, Hogben & Roach, 2006). Thus the larger frequency deviant used in the oddball paradigm (200 Hz) would have been readily detectable by all participants. The *a priori* prediction was therefore made that insofar as indices of mismatch responses related to behavioural discrimination, this relationship should be seen for the smaller frequency deviant (30 Hz). A *p*-value of .05/8 = .006 was adopted to adjust for the multiple comparisons. The relevant correlations are shown in Table 2, both before and after partialling out the effect of age group. The only correlation with FD threshold that was significant was with the early ITC index for the small deviant. This remained significant after age group was partialled out.

**Table 2:** Pearson correlations between FD threshold and MMN indices, (A) = raw correlation, (B) = with age group partialled out, *N* = 83

	A	B
Large deviant, MMN mean amplitude	−.105	−.181
Large deviant, early ITC	−.117	−.058
Large deviant, LDN mean amplitude	−.058	−.059
Large deviant, late ITC	.130	.078
Small deviant, MMN mean amplitude	.228	.171
Small deviant, early ITC	−.385*	−.385*
Small deviant, LDN mean amplitude	.049	.051
Small deviant, late ITC	−.073	−.091

* *p*-value < .006.

## Discussion

### Development of mismatch responses

When traditional averaging methods were used to evaluate the MMN, we found an increase in amplitude with age. These results appear to conflict with previous reports that MMN in children is equivalent to or larger than that found in adults (e.g. Gomot, Giard, Roux, Barthelemy & Bruneau 2000; Kraus, Koch, McGee, Nicol & Cunningham, 1999; Kraus *et al*., 1993; Shafer, Morr, Kreuzer & Kurtzberg, 2000). However, developmental trends may have been obscured in these studies because the measurement of MMN would have been affected by the greater ERP variance found in children as compared with adults, which could lead to spuriously large peaks. We avoided this problem by assessing MMN in terms of mean amplitude over a specified window. To check our concerns about bias introduced by peak amplitude, we also re-ran the analysis of tone MMNs using peak rather than mean amplitude, and including the dummy condition. This gave a significantly larger MMN in the youngest group compared to the other two groups for the *dummy* condition (see Supplementary material). Our findings clearly demonstrate the problems that result when comparing peak amplitudes for two groups differing in variance: since standards and dummy ‘deviants’ were the same in this condition, the larger mismatch seen for children must simply be noise.

### Spectral characteristics of mismatch responses

Time-frequency analysis throws light on what it is that changes with age, and illustrates the limitations of relying on traditional averaging methods. Perhaps the most striking finding from this study is a null result – the failure to see any difference in the power spectrum between genuine MMN responses and dummy responses in the theta frequency bands, despite clear differences in their mean amplitudes. There was only the faintest hint of a change in ERSP in mismatch files for adults around 100 ms. Previous work on auditory ERPs has shown that the onset of a stimulus is associated with both phase resetting and an increase in power in the theta range (Fuentemilla *et al*., 2006). However, for difference waves based on deviants, our results agree with those of Bishop and Hardiman (2010) in showing that phase resetting in the theta frequency band rather than a phasic increase in power is the primary mechanism by which the MMN is generated. It should be noted, however, that the baseline and epoch length used in our studies was restricted by our use of an SOA of 1000 ms to 200 ms, and may have been too short for reliable identification of ERSP at low frequencies: in contrast, Fuentemilla *et al*. (2008) and Yordanova and Kolev (2008) both used a baseline of 1000 ms.

More anecdotally, it is worth noting that our original plan had been to conduct only conventional time-domain analysis of difference waves. We used SOBI rather than another form of independent component analysis with the initial aim of identifying and removing components corresponding to the regular oscillations that were visible in the raw EEG after subtraction of the average standard wave (see Supplementary material). This proved totally unsatisfactory because removal of the oscillations also removed the MMN in the averaged waveform; this indicated that, far from being artefacts, the oscillations were key elements of the mismatch response.

Theta synchronization has been investigated previously in the context of memory processes: animal studies have indicated that the hippocampus is a source of theta oscillations, and increased synchronization of theta activity has been noted to accompany declarative learning (Sauseng & Klimesch, 2008). The findings from the current study suggest that theta synchronization also indexes change detection over much briefer intervals when auditory sensory memory for a standard sound is compared with an incoming sound.

The results from single-trial analysis show good agreement with a series of studies summarized by Yordanava and Kolev (2008), who used an auditory oddball paradigm to compare P300 responses in children and adults. In their study, participants were asked to count 25 deviant tones (800 Hz) occurring among 75 standard tones (1200 Hz). In another comparison condition, 50 deviant tones were presented alone. Despite substantial differences between their paradigm and the one used here (number of trials, inter-trial interval, deviant frequency, attentional demands, and analysis of original trials vs. trials with standard subtracted), developmental trends were consistent with current data in showing that phase-locking at the theta frequency was higher in adults than children during the first 300 ms after stimulus onset. Furthermore, adults showed more phase-locking in the first 300 ms post-stimulus onset than later in the epoch, whereas in young children the amount of phase-locking was greater during a later interval from 300 to 600 ms post-onset. Yordanova and Kolev (2008) related these findings to the late maturation of the frontal lobes. Our results indicate that phase-locked theta is not, as sometimes thought, only seen with focused attention, but rather reflects automatic change detection, since it is prominent in difference waves from a classic mismatch paradigm with no active attention to stimuli (see also Mülller *et al*., 2009; Shahin *et al*., 2010).

When analysed in terms of ITC, the data confirm that the MMN continues to develop through childhood and adolescence. Furthermore, the extent of phase-locking in the MMN interval with a small frequency deviant was the only measure that was predictive of behavioural frequency discrimination threshold. There has been much discussion in the literature on development of auditory discrimination as to whether high thresholds in children are due to nonsensory factors or whether they reflect variability in the neural representation of auditory features (e.g. Buss, Hall & Grose, 2009). The reduced phase synchronization that we observed in children in a paradigm with no task demands would seem to provide a potential measure of the latter mechanism, which has been referred to as ‘internal noise’.

Although it is tempting to conclude that the observed changes are due to intrinsic maturational processes, they could also reflect experience-dependent learning. In this regard, a study by Shahin, Roberts, Chau, Trainor and Miller (2008) is of particular interest. They showed that a measure of high-frequency oscillations, induced gamma-band activity, to piano tone stimuli increased in young children after 1 year of piano training, whereas control children with no music lessons showed no effect. There would be considerable interest in doing parallel studies of the effects of musical training on the lower-frequency oscillations that are the subject of the current study, and also to consider how familiarity with specific speech sounds affects phase synchronization of theta responses to sound changes (cf. Näätänen, Lehtokoski, Lennes, Cheour, Huotilainen, Ilvonen, Vainio, Alku, Ilmoniemi, Luuk, Allik, Sinkkonen & Alho, 1997).

### The Late Discriminative Negativity

A supplementary goal of our study was to consider further the characteristics of the LDN, which has received less research attention than the MMN. There are two characteristics of the LDN that have been described in the literature: it is greater in children than in adults, and more pronounced for speech than for nonspeech signals. Both findings were obtained in the current dataset. Nevertheless, the speech–nonspeech comparison needs to be treated with caution, as the stimuli contrasted in important respects other than ‘speechness’. Acoustically, the speech stimuli were more complex, and also the acoustic differences between standards and deviants involved a number of acoustic changes, whereas tones differed only in frequency. In addition, with hindsight one can see that, while the speech and nonspeech conditions both involved two deviants, they differed in the extent to which they conformed to the optimal paradigm described by Näätänen, Pakarinen, Rinne and Takegata (2004). These authors showed that it is possible to get large mismatch responses while including multiple deviants in an oddball design provided each deviant differs from the standard on only one dimension. This was the case for the syllables used here, where deviant 1 had the same consonant as the standard, and deviant 2 had the same vowel. This means that each deviant could strengthen the representation of one part of the standard, and in effect the presentation of both the consonant and the vowel portion of the standard occurred on 85% of trials. For tone stimuli this was not the case, because both deviants differed on the same dimension, frequency, and the standard frequency occurred on only 70% of trials. When viewed from the perspective of stimulus dimensions, rather than individual stimuli, differences between speech and nonspeech conditions are confounded with differences in deviant probability. Further experiments are needed to determine how far this affects mismatch responses at different latencies.

One intriguing and novel observation about the LDN is that it was reliably larger for small than for large deviants. This is the opposite pattern to that seen for the MMN, and agrees with other sources of evidence (Čeponiene, Lepistö, Soininen, Aronen, Alku & Näätänen, 2004) that this component should not be regarded as a late manifestation of the MMN. It may instead reflect additional processing of auditory stimuli that occurs when the salient features of the stimulus are hard to detect, or, given the age effects on LDN, when the listener has less experience of such stimuli.

### Methodological points

Our analysis differed from that often used in developmental studies of mismatch responses in several respects in addition to the use of time-frequency analysis, two of which are worthy of specific comment. First, we simplified data analysis by deriving a spatial principal component from the overall auditory ERP mean for each participant and using its weightings to create a new channel in the dataset. Previously we had adopted the more traditional approach of either focusing on a single electrode at which activity was maximal, typically Fz, or of entering data from a set of fronto-central electrodes into a multivariate analysis. Reliance on a single electrode has the disadvantage that it ignores a large proportion of the data, and may be misleading if the topography of the response changes with age. But multivariate analysis with electrode as a repeated measure is fraught with statistical problems because of the strong interdependence of results from closely located electrodes: according to Field (2005), application of repeated measures ANOVA is problematic when there are strong violations of sphericity assumptions. Field (2005) noted that MANOVA avoids problems of sphericity violation, but at the cost of reduced power to detect genuine effects. The method used here, of extracting a spatial principal component from the whole dataset, appears more satisfactory because it uses all the data but allows one to use simpler univariate analysis to compare groups and conditions. A potential concern is that contributions from specific electrodes might vary in different individuals, but that can be tested by plotting the topography of component weights, as shown in Figure 1.

A second feature of our analysis is the use of dummy as well as genuine mismatch difference waves to establish the validity and reliability of findings. This approach is not new: it was used by McGee, Kraus and Nicol (1997) in their comparison of different methods for measuring the MMN, and was also recommended by Picton and Taylor (2007). However, it has been used only rarely in subsequent studies concerned with developmental or clinical group comparisons. We have found this method valuable for giving confidence in findings in a field where conventional statistical approaches are often problematic, because of interdependence of data points in time and space, and because of difficulties in specifying *a priori* which time window or electrode to analyse.

## Conclusions

Using tone stimuli, we found converging evidence for prolonged development of auditory discrimination using both behavioural and electrophysiological measures. The effects were clearest when MMN was analysed using a measure of ITC that assessed the extent to which the phase of theta rhythms was reset by occurrence of a deviant tone. This showed clear developmental trends, and also correlated with frequency discrimination thresholds. For speech stimuli, we did not have data on behavioural discrimination, but we were able to demonstrate similar findings with electrophysiological measures, with ITC again providing evidence that the MMN is the result of theta phase resetting by a deviant stimulus. These findings join the growing body of evidence showing that brain mechanisms underlying auditory processing continue to develop up to adulthood.

## Supporting information

**Table S1** Anova: Mean amplitude, 100–250 ms

**Table S2** Anova: Mean amplitude, 300–550 ms

**Table S3** Anova: Peak Latency, 100–250 ms

**Table S4** Anova: Peak Latency, 300–550 ms

**Table S5** Anova: ITC, 0–300 ms

**Table S6** Anova: ITC, 300–600 ms

**Table S7** Anova: ERSP, 0–300 ms

**Table S8** Anova: ERSP, 300–600 ms

**Table S9** Anova: Peak amplitude, 100 = 250 ms, including dummy deviants

**Figure S1** Illustrative continuous files showing oscillations in difference waves

## References

[b1] Banai K, Ahissar M (2006). Auditory processing deficits in dyslexia: task or stimulus related?. Cerebral Cortex.

[b2] Başar E, Başar-Eroglu C, Karakas S, Schürmann M (1999). Oscillatory brain theory: a new trend in neuroscience. IEEE Engineering in Medicine and Biology.

[b3] Başar E, Başar-Eroglu C, Parnefjord R, Rahn E, Schürmann M, Başar E, Bullock TH (1992). Evoked potentials: ensembles of brain induced rhythmicities in the alpha, theta and gamma ranges. Induced rhythms in the brain.

[b4] Bishop DVM (2003). The Test for Reception of Grammar, version 2 (TROG-2).

[b5] Bishop DVM, Hardiman MJ (2010). Measurement of mismatch negativity in individuals: a study using single-trial analysis. Psychophysiology.

[b6] Bishop DVM, Hardiman M, Uwer R, von Suchodoletz W (2007). Maturation of the long-latency auditory ERP: step function changes at start and end of adolescence. Developmental Science.

[b7] Boersma P, Weenink D (2005). Praat: a system for doing phonetics by computer. Glot International.

[b8] Buss E, Hall JW, Grose JH (2009). Psychometric functions for pure tone intensity discrimination: slope differences in school-aged children and adults. Journal of the Acoustical Society of America.

[b9] Čeponiené R, Lepistö T, Soininen M, Aronen E, Alku P, Näätänen R (2004). Event-related potentials associated with sound discrimination versus novelty detection in children. Psychophysiology.

[b10] Cheour M, Korpilahti P, Martynova O, Lang AH (2001). Mismatch negativity and late discriminative negativity in investigating speech perception and learning in children and infants. Audiology and Neuro-Otology.

[b11] Cheour M, Leppänen PH, Kraus N (2000). Mismatch negativity (MMN) as a tool for investigating auditory discrimination and sensory memory in infants and children. Clinical Neurophysiology.

[b12] Craig C (2008). GoldWave (Version 5.23).

[b13] Delorme A, Makeig S (2004). EEGLAB: an open source toolbox for analysis of single-trial EEG dynamics including independent component analysis (sccn.ucsd.edu/eeglab/). Journal of Neuroscience Methods.

[b14] Eggermont JJ, Ponton CW (2003). Auditory-evoked potential studies of cortical maturation in normal hearing and implanted children: correlations with changes in structure and speech perception. Acta Otolaryngologica.

[b15] Field A (2005). Discovering statistics using SPSS.

[b16] Findlay JM (1978). Estimates on probability functions: a more virulent PEST. Perception and Psychophysics.

[b17] Fuentemilla L, Marco-Pallarés J, Grau C (2006). Modulation of spectral power and phase resetting of EEG contributes differentially to the generation of auditory event-related potentials. NeuroImage.

[b18] Fuentemilla L, Marco-Pallarés J, Münte TF, Grau C (2008). Theta EEG oscillatory activity and auditory change detection. Brain Research.

[b19] Gomot M, Giard MH, Roux S, Barthelemy C, Bruneau N (2000). Maturation of frontal and temporal components of mismatch negativity (MMN) in children. NeuroReport.

[b20] Guthrie D, Buchwald J (1991). Significance testing of difference potentials. Psychophysiology.

[b21] Halliday L, Bishop DVM (2005). Frequency discrimination and literacy skills in children with mild to moderate sensorineural hearing loss. Journal of Speech, Language and Hearing Research.

[b22] Heath SM, Bishop DVM, Hogben JH, Roach NW (2006). Psychophysical indices of perceptual functioning in dyslexia: a psychometric analysis. Cognitive Neuropsychology.

[b23] Hsiao FJ, Wu ZA, Ho LT, Lin YY (2009). Theta oscillation during auditory change detection: an MEG study. Biological Psychology.

[b24] Illing R (2004). Maturation and plasticity of the central auditory system. Acta Otolaryngol Suppl..

[b25] Kolev V, Rosso OA, Yordanova J (2001). A transient dominance of theta ERP component characterizes passive auditory processing: evidence from a developmental study. NeuroReport.

[b26] Korpilahti P, Krause CM, Holopainen IE, Lang AH (2001). Early and late mismatch negativity elicited by words and speech-like stimuli in children. Brain and Language.

[b27] Kraus N, Koch DB, McGee TJ, Nicol TG, Cunningham J (1999). Speech–sound discrimination in school-age children: psychophysical and neurophysiological measures. Journal of Speech, Language, and Hearing Research.

[b28] Kraus N, McGee T, Carrell T, Sharma A, Micco A, Nicol T (1993). Speech-evoked cortical potentials in children. Journal of the American Academy of Audiology.

[b29] Lenroot RK, Schmitt JE, Ordaz SJ, Wallace GL, Neale MC, Lerch JP, Kendler KS, Evans AC, Giedd JN (2009). Differences in genetic and environmental influences on the human cerebral cortex associated with development during childhood and adolescence. Human Brain Mapping.

[b30] McGee T, Kraus N, Nicol T (1997). Is it really a mismatch negativity? An assessment of methods for determining response validity in individual subjects. Electroencephalography and Clinical Neurophysiology.

[b31] Makeig S (1993). Auditory event-related dynamics of the EEG spectrum and effects of exposure to tones. Electroencephalography and Clinical Neurophysiology.

[b32] Makeig S, Debener S, Onton J, Delorme A (2004). Mining event-related brain dynamics. Trends in Cognitive Sciences.

[b33] Moore DR (2002). Auditory development and the role of experience. British Medical Bulletin.

[b34] Moore JK, Guan Y (2001). Cytoarchitectural and axonal maturation in human auditory cortex. Journal of the Association for Research in Otolaryngology.

[b35] Moore JK, Linthicum FH (2007). The human auditory system: a timeline of development. International Journal of Audiology.

[b36] Mülller V, Gruber W, Klimesch W, Lindenberger U (2009). Lifespan differences in cortical dynamics of auditory perception. Developmental Science.

[b37] Näätänen R, Lehtokoski A, Lennes M, Cheour M, Huotilainen M, Ilvonen A, Vainio M, Alku P, Ilmoniemi RJ, Luuk A, Allik J, Sinkkonen J, Alho K (1997). Language-specific phoneme representations revealed by electric and magnetic brain responses. Nature.

[b38] Näätänen R, Paavilainen P, Rinne T, Alho K (2007). The mismatch negativity (MMN) in basic research of central auditory processing: a review. Clinical Neurophysiology.

[b39] Näätänen R, Pakarinen S, Rinne T, Takegata R (2004). The mismatch negativity (MMN): towards the optimal paradigm. Clinical Neurophysiology.

[b40] Oades RD, Dittmann-Balcar A, Zerbin D (1997). Development and topography of auditory event-related potentials (ERPs): mismatch and processing negativity in individuals 8–22 years of age. Psychophysiology.

[b41] Picton TW, Taylor MJ (2007). Electrophysiological evaluation of human brain development. Developmental Neuropsychology.

[b42] Sauseng P, Klimesch W (2008). What does phase information of oscillatory brain activity tell us about cognitive processes?. Neuroscience and Biobehavioral Reviews.

[b43] Sayers BM, Beagley HA, Henshall WR (1974). The mechanism of auditory evoked EEG responses. Nature.

[b44] Schürmann M, Başar E (1994). Topography of alpha and theta oscillations upon auditory and visual stimuli in humans. Biological Cybernetics.

[b45] Shafer VL, Morr ML, Kreuzer JA, Kurtzberg D (2000). Maturation of mismatch negativity in school-aged children. Ear and Hearing.

[b46] Shahin AJ, Roberts LE, Chau W, Trainor LJ, Miller LM (2008). Music training leads to the development of timbre-specific gamma band activity. NeuroImage.

[b47] Shahin AJ, Trainor LJ, Roberts LE, Backer KC, Miller LM (2010). Development of auditory phase-locked activity for music sounds. Journal of Neurophysiology.

[b48] Sutcliffe P, Bishop DVM (2005). Psychophysical design influences frequency discrimination performance in young children. Journal of Experimental Child Psychology.

[b49] Sutcliffe P, Bishop D, Houghton S, Taylor M (2006). Effect of attentional state on frequency discrimination: a comparison of children with ADHD on and off medication. Journal of Speech, Language and Hearing Research.

[b50] Tang AC, Sutherland MT, McKinney CJ (2005). Validation of SOBI components from high-density EEG. NeuroImage.

[b51] Torgesen JK, Wagner R, Rashotte C (1999). Test of Word Reading Efficiency (TOWRE).

[b52] Uwer R, Albrecht R, von Suchodoletz W (2002). Automatic processing of tones and speech stimuli in children with specific language impairment. Developmental Medicine and Child Neurology.

[b53] Wechsler D (1999). Wechsler Abbreviated Scale of Intelligence.

[b54] Werner LA, Marean GC (1996). Human auditory development.

[b55] Whitford TJ, Rennie CJ, Grieve SM, Clark CR, Gordon E, Williams LM (2007). Brain maturation in adolescence: concurrent changes in neuroanatomy and neurophysiology. Human Brain Mapping.

[b56] Yordanova J, Kolev V, Schmidt LA, Segalowitz SJ (2008). Event-related brain oscillations in normal development. Developmental psychophysiology: Theory, systems and methods.

