# Supplementary material for: Is auditory discrimination mature by middle childhood? A study using time-frequency analysis of mismatch responses from 7 years to adulthood
Source: Dev Sci. 2011 Mar;14(2):402–16. doi: 10.1111/j.1467-7687.2010.00990.x (PMC3083517; doi:10.1111/j.1467-7687.2010.00990.x)
Supplement: Supplementary file 1 [file desc0014-0402-SD1.ppt]

## Slide 1
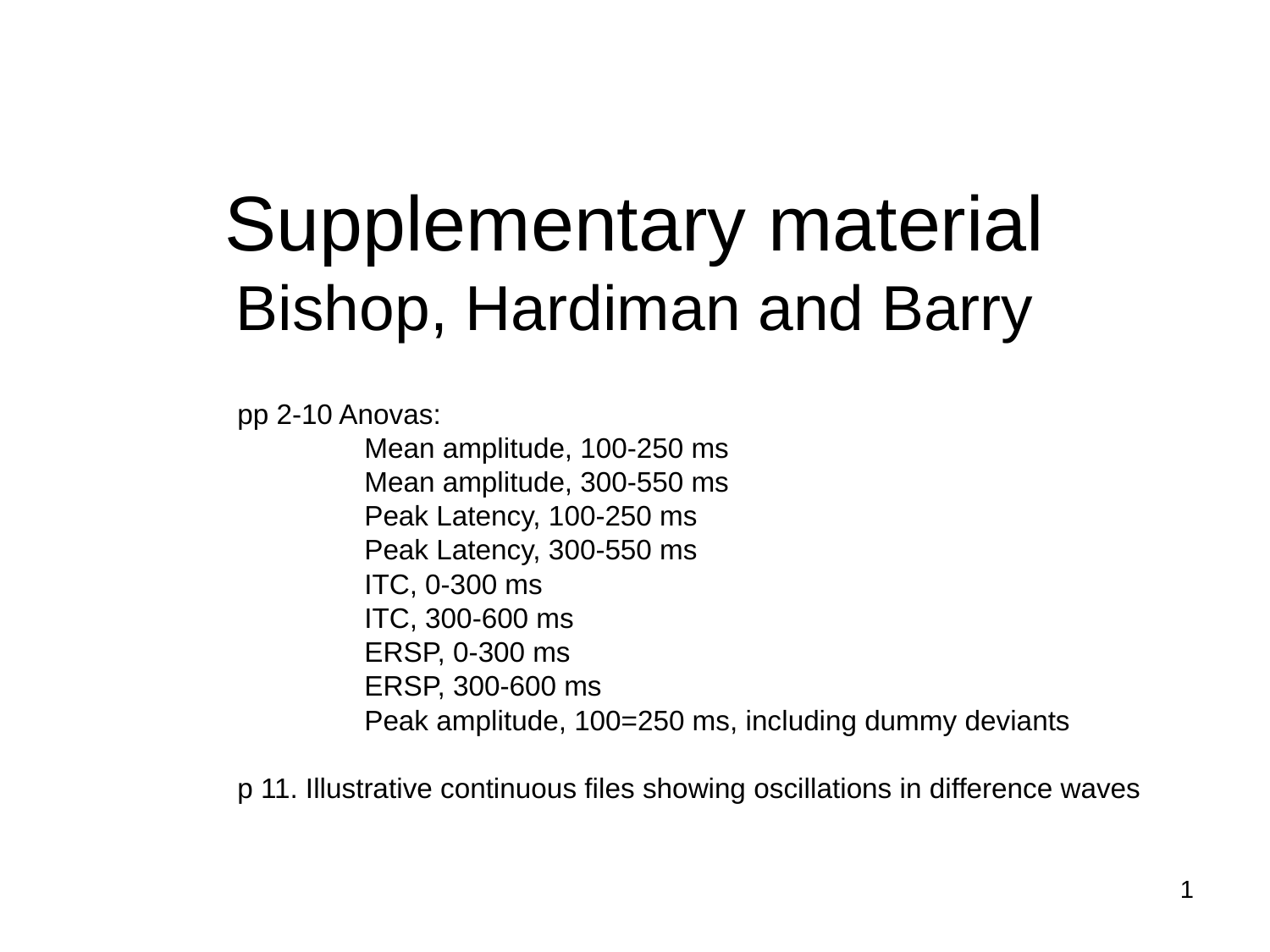

# Supplementary material Bishop, Hardiman and Barry
pp 2-10 Anovas:
	Mean amplitude, 100-250 ms
	Mean amplitude, 300-550 ms
	Peak Latency, 100-250 ms
	Peak Latency, 300-550 ms
	ITC, 0-300 ms
	ITC, 300-600 ms
	ERSP, 0-300 ms
	ERSP, 300-600 ms
	Peak amplitude, 100=250 ms, including dummy deviants
p 11. Illustrative continuous files showing oscillations in difference waves
<number>

## Slide 2
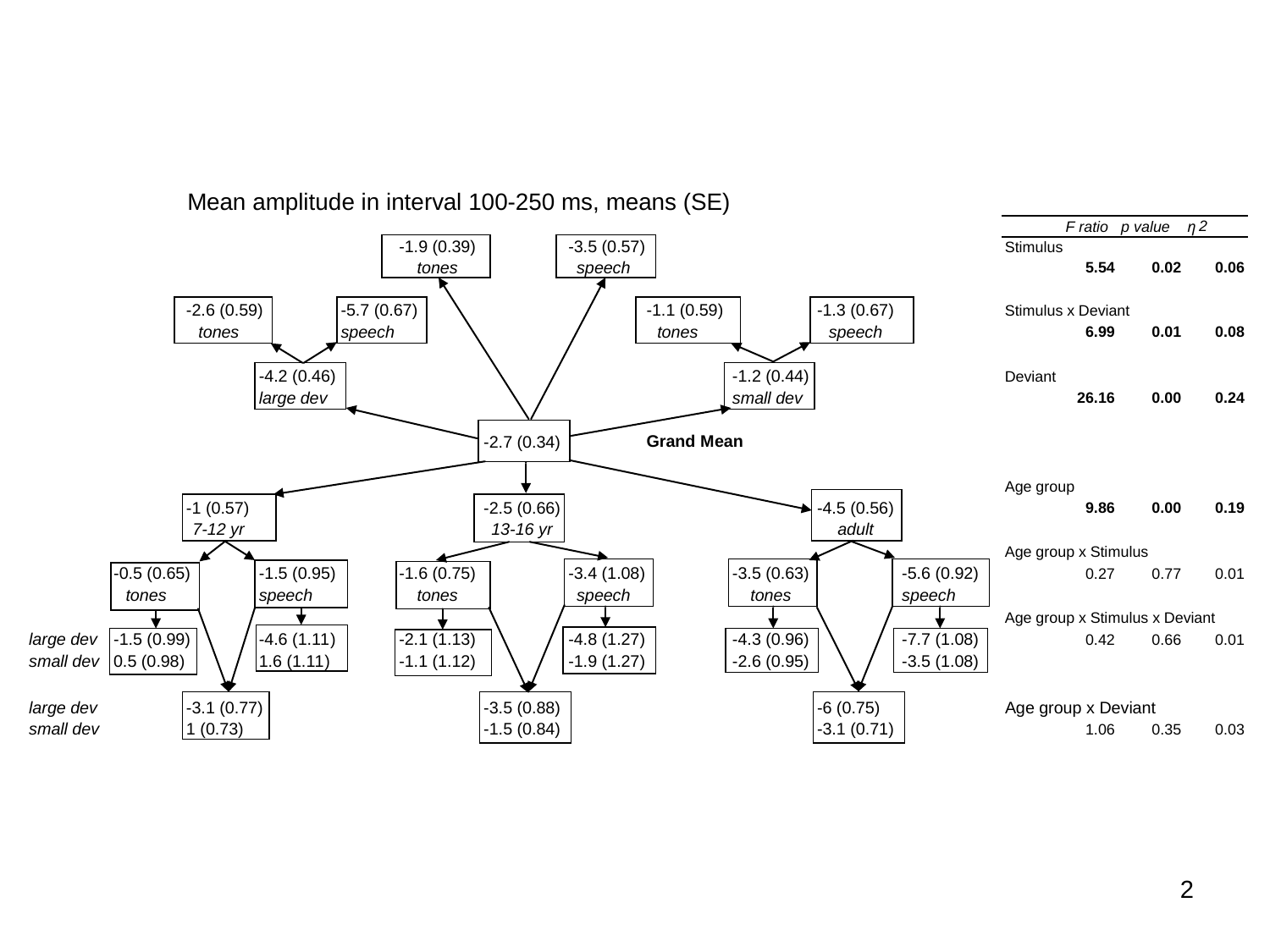

<number>

## Slide 3
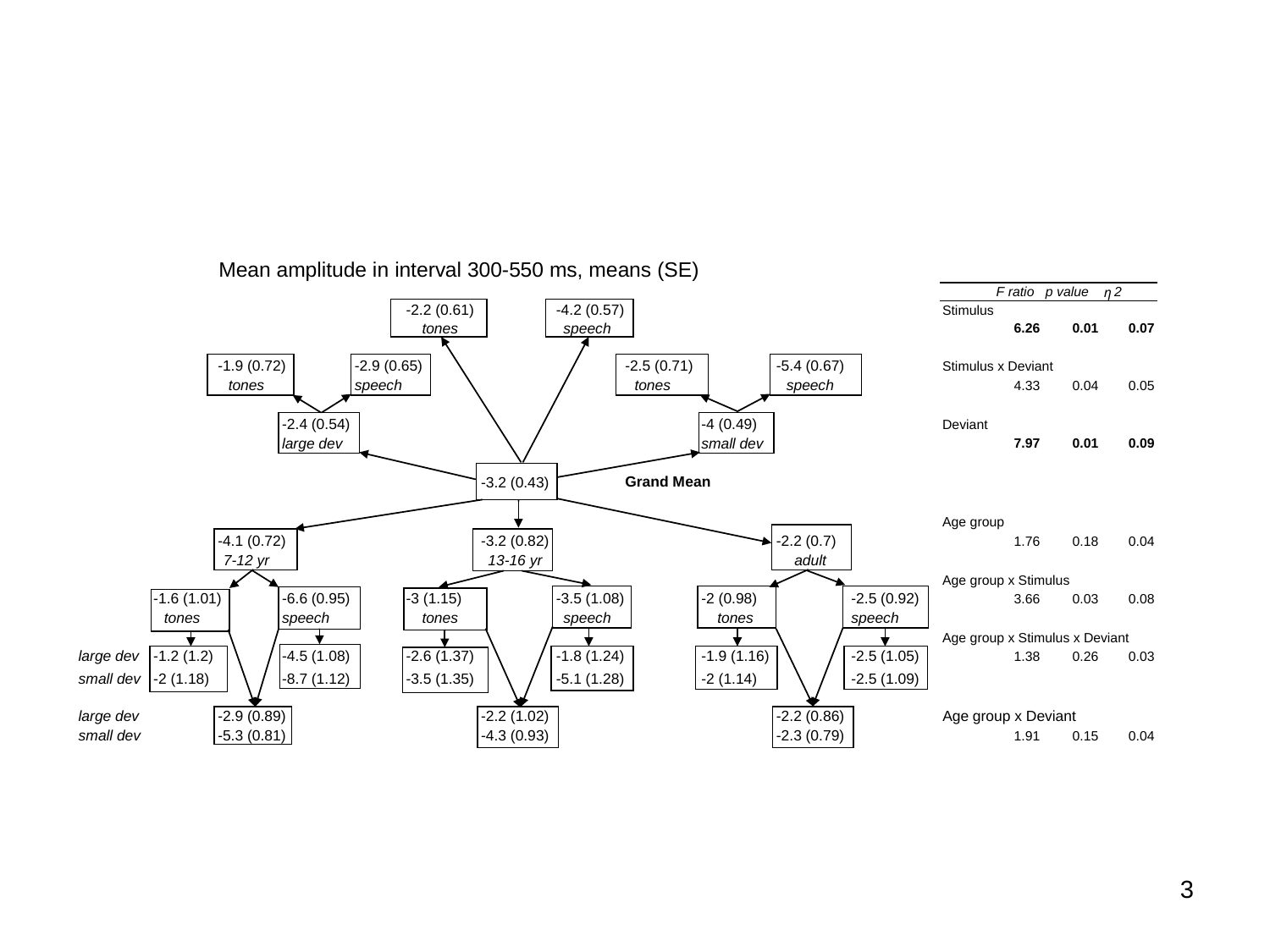

<number>

## Slide 4
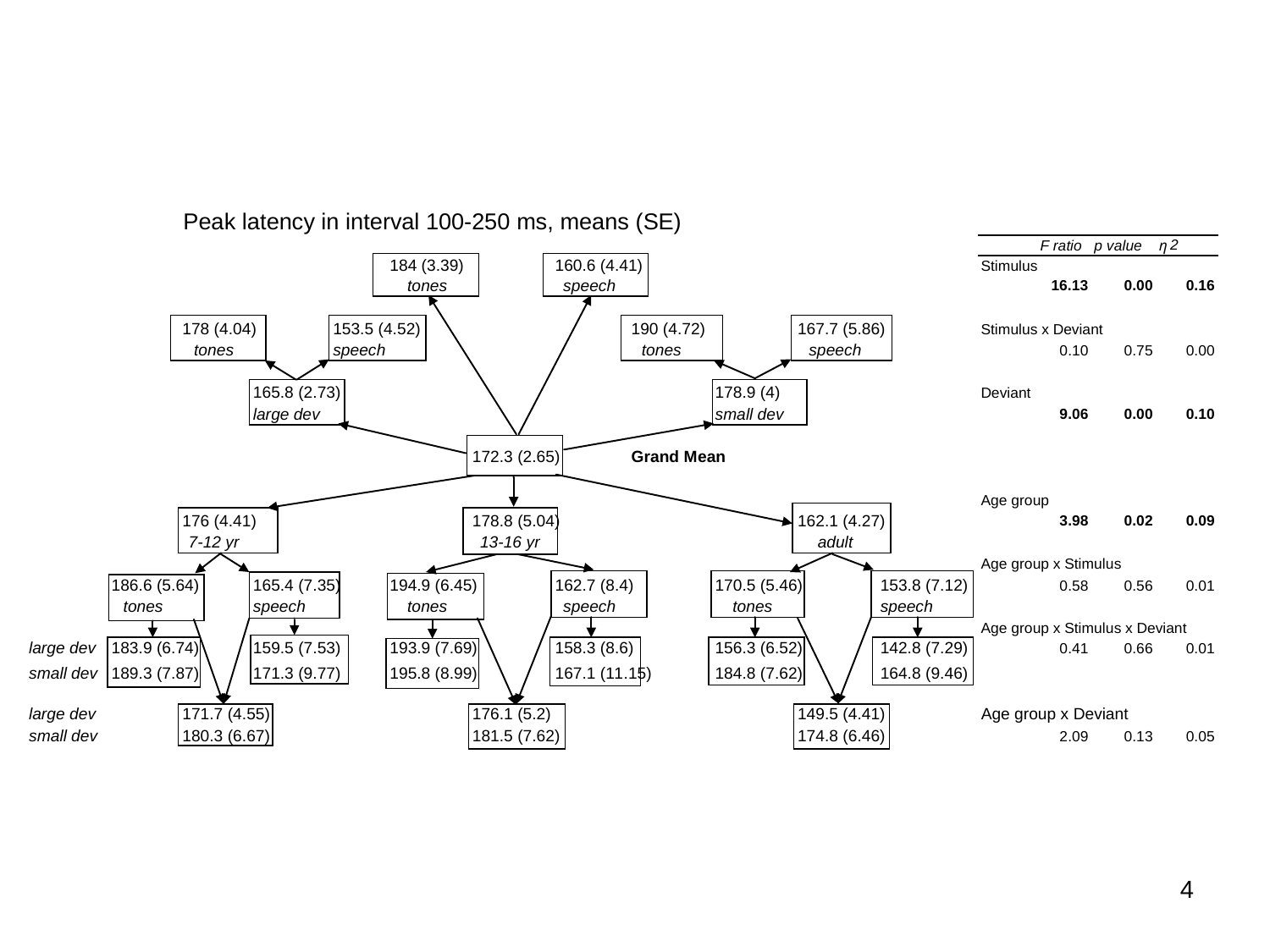

<number>

## Slide 5
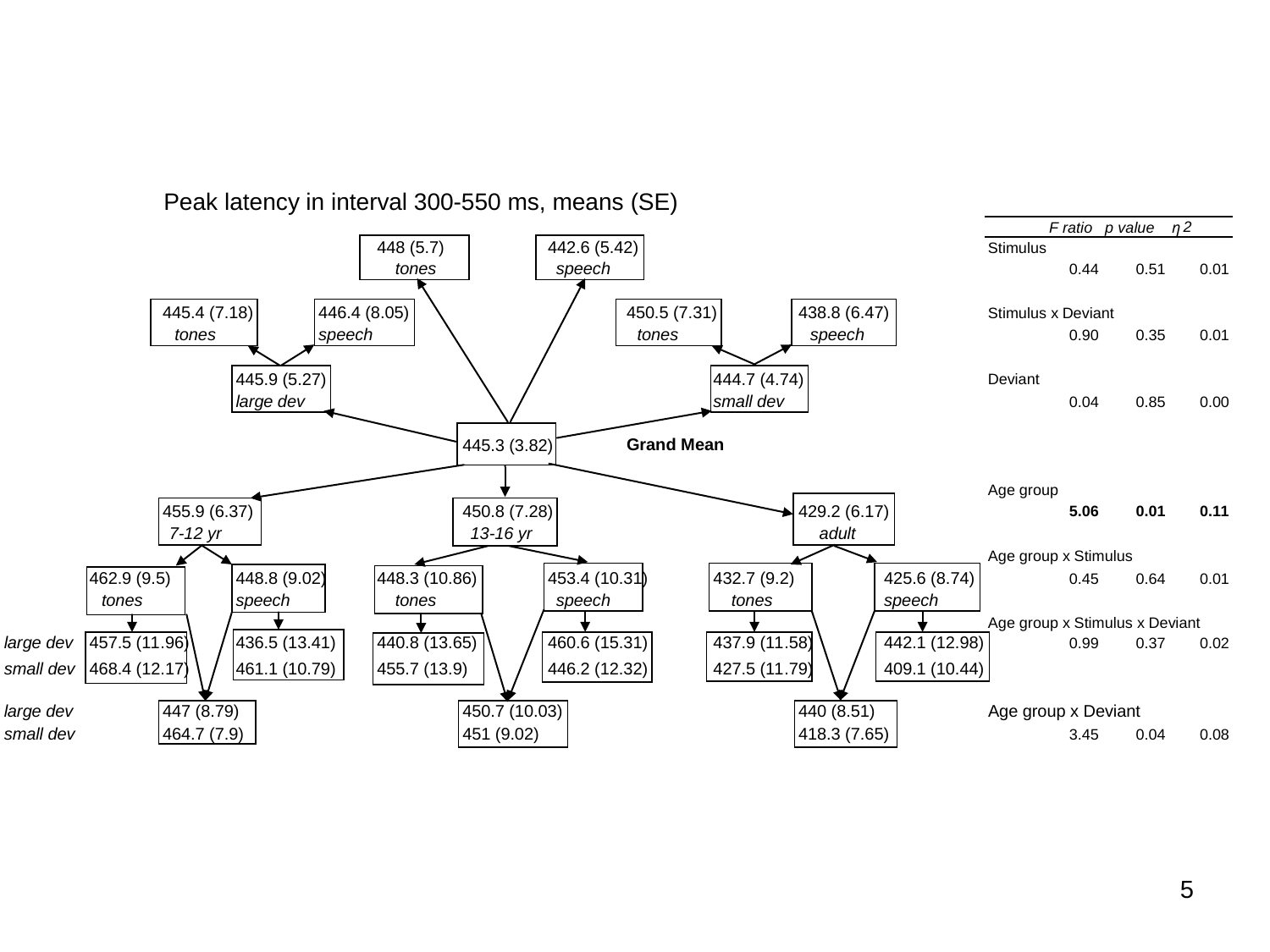

<number>

## Slide 6
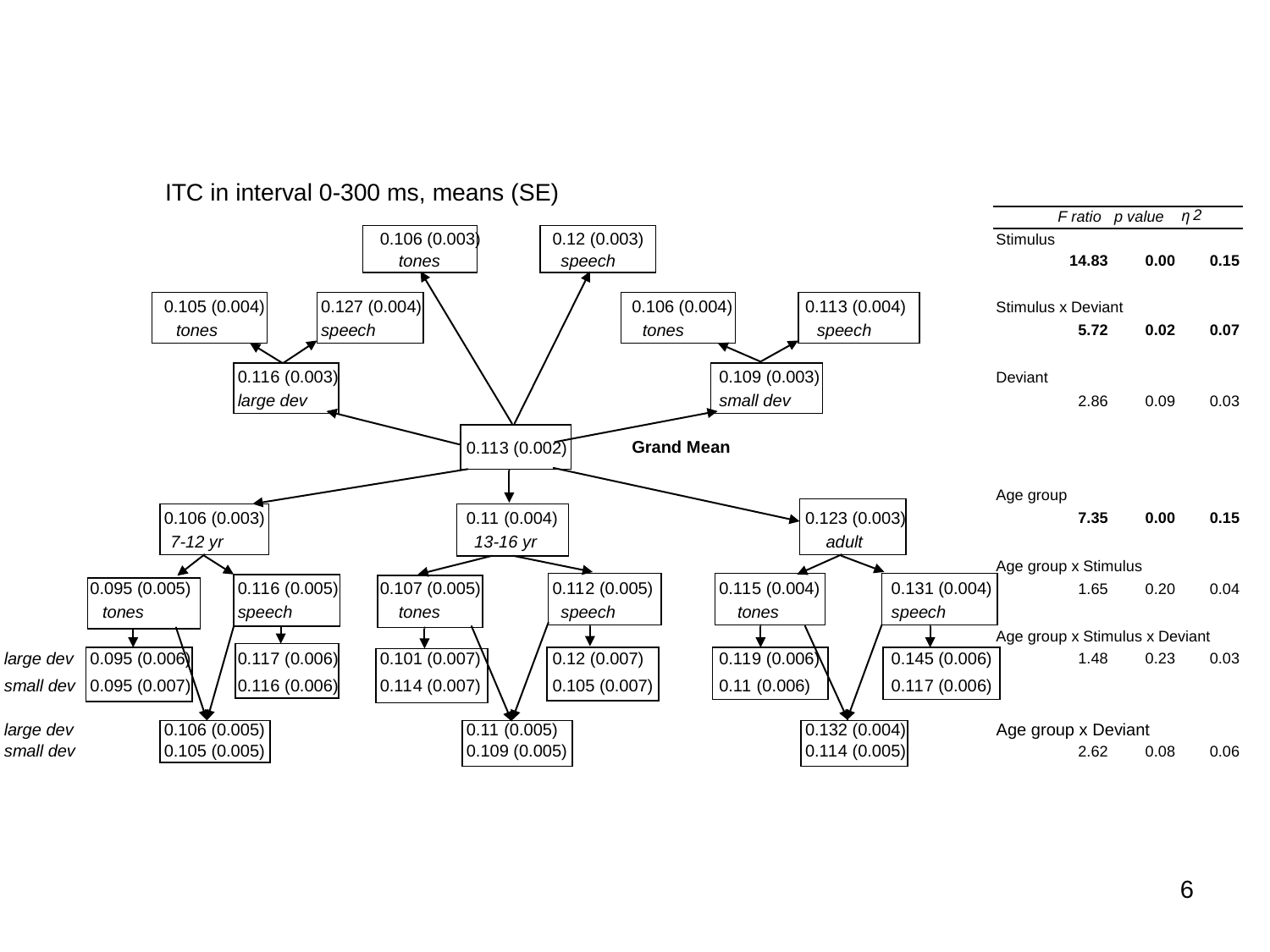

<number>

## Slide 7
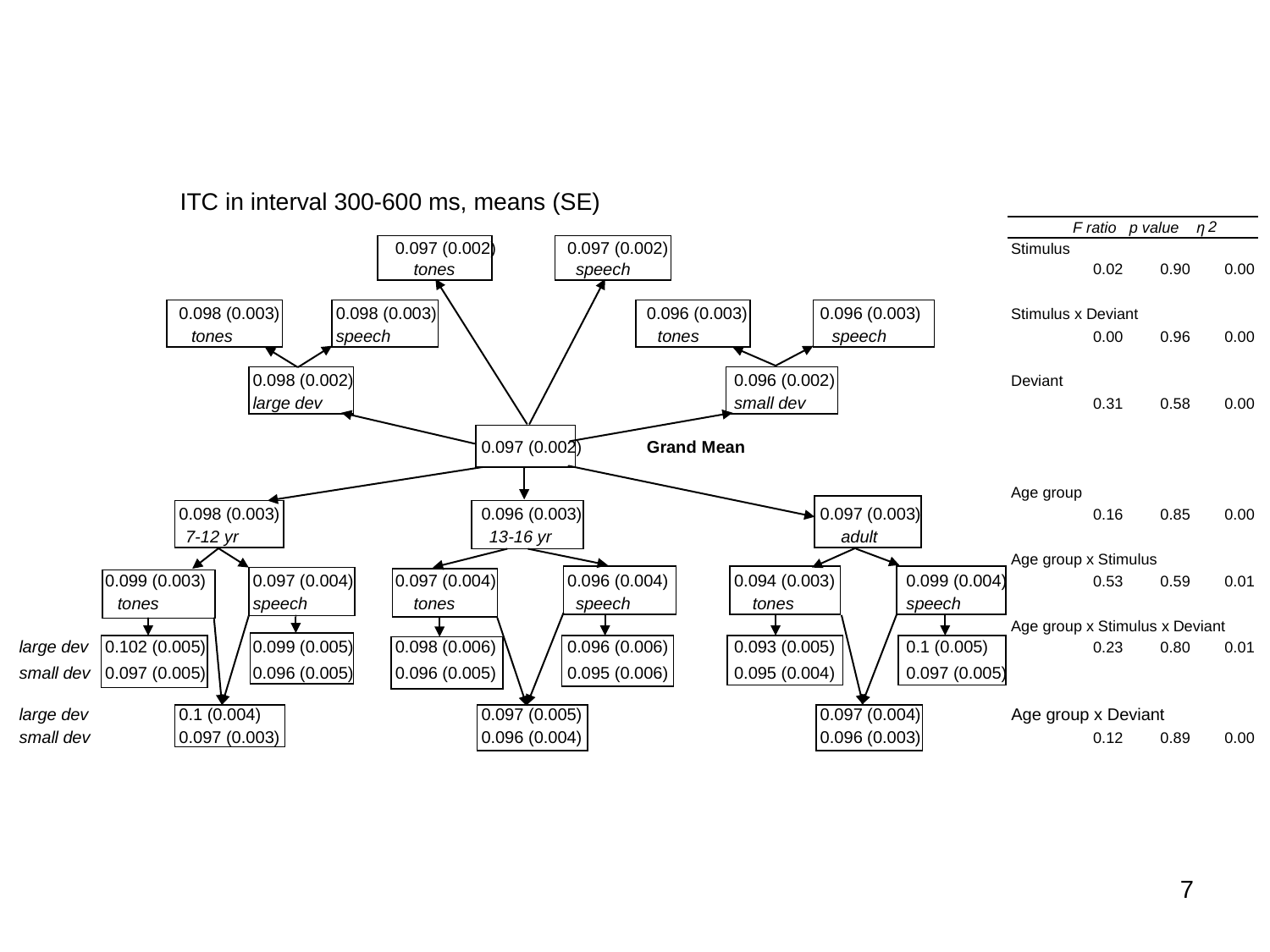

<number>

## Slide 8
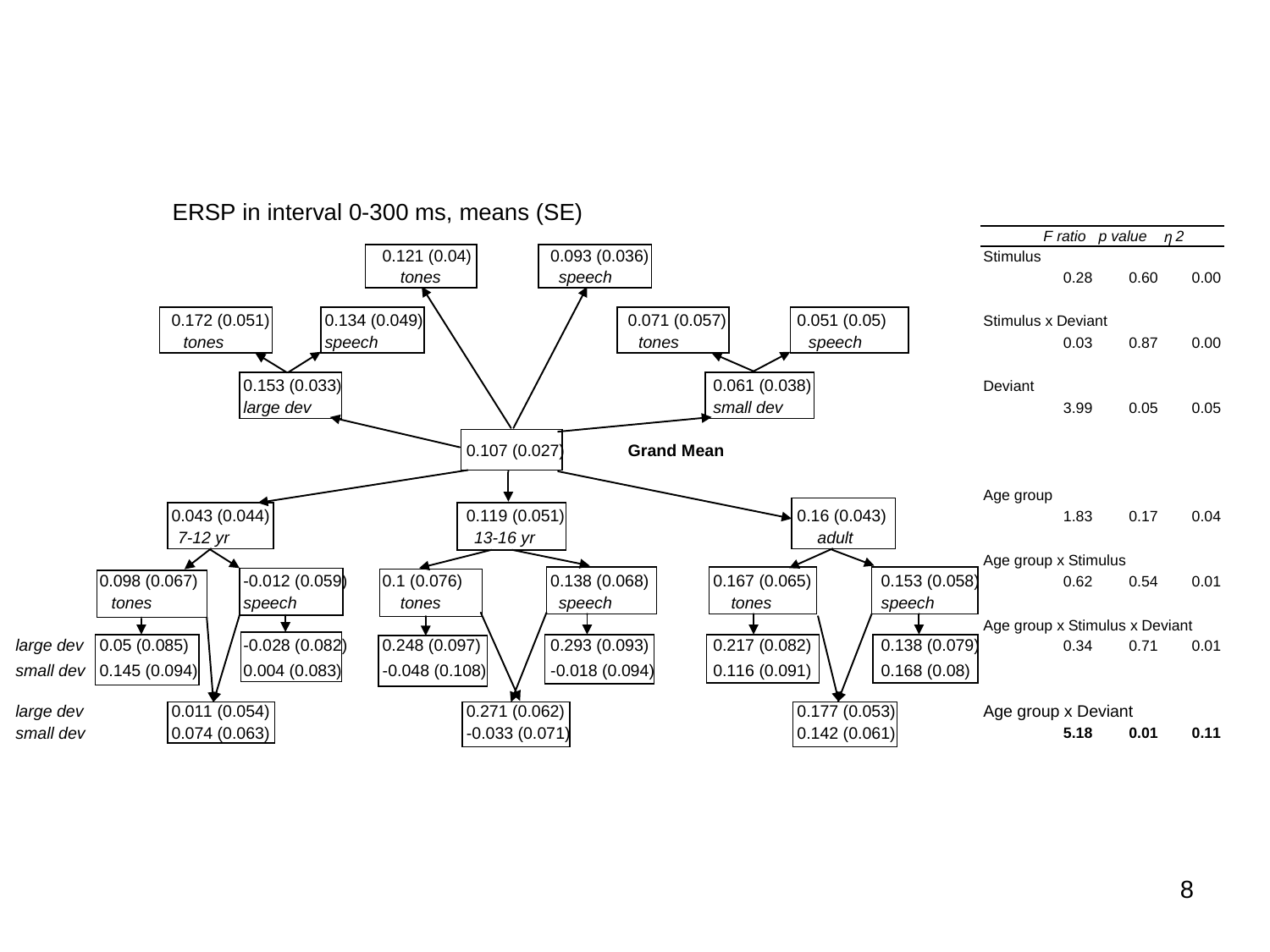

<number>

## Slide 9
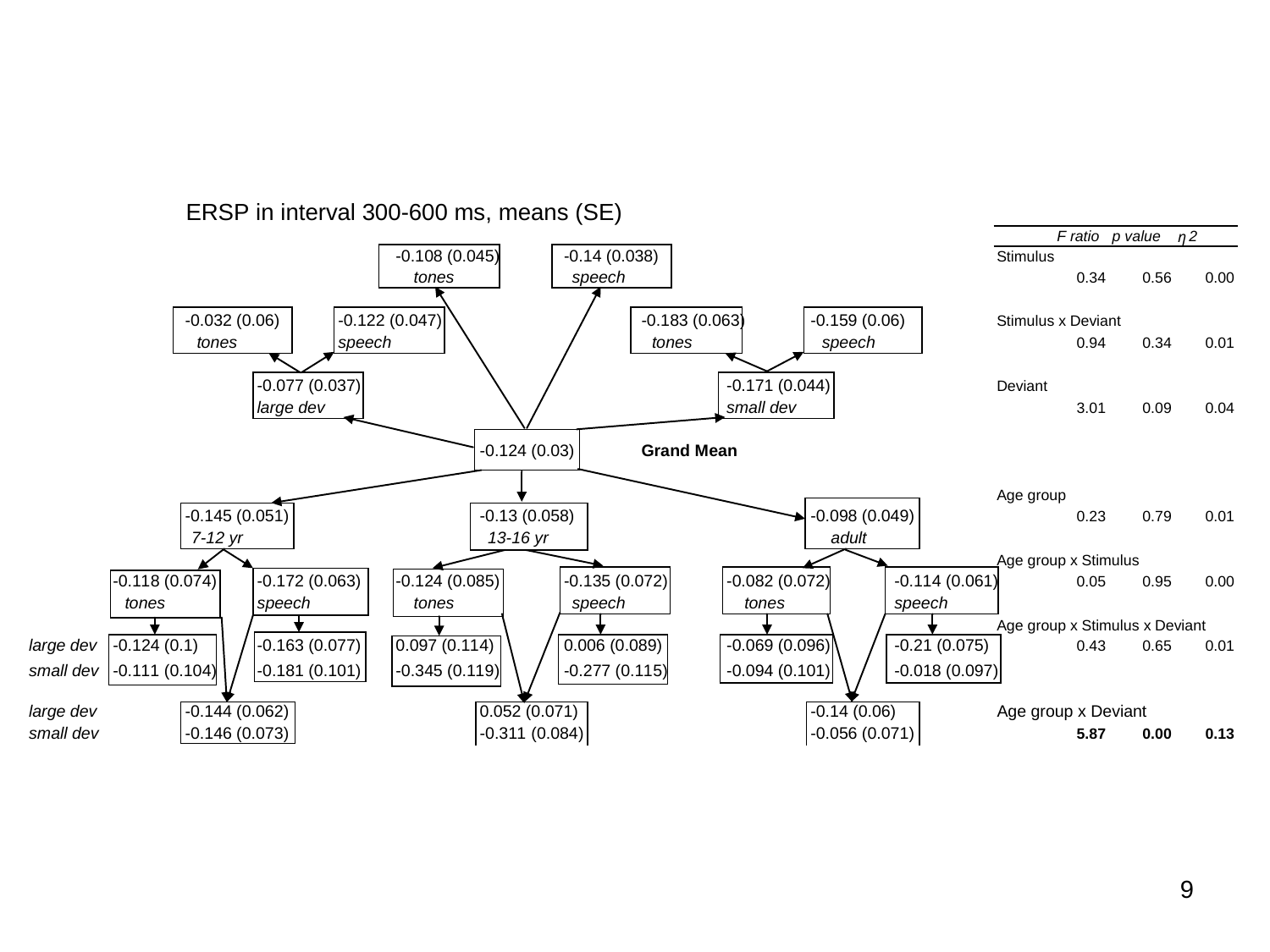

<number>

## Slide 10
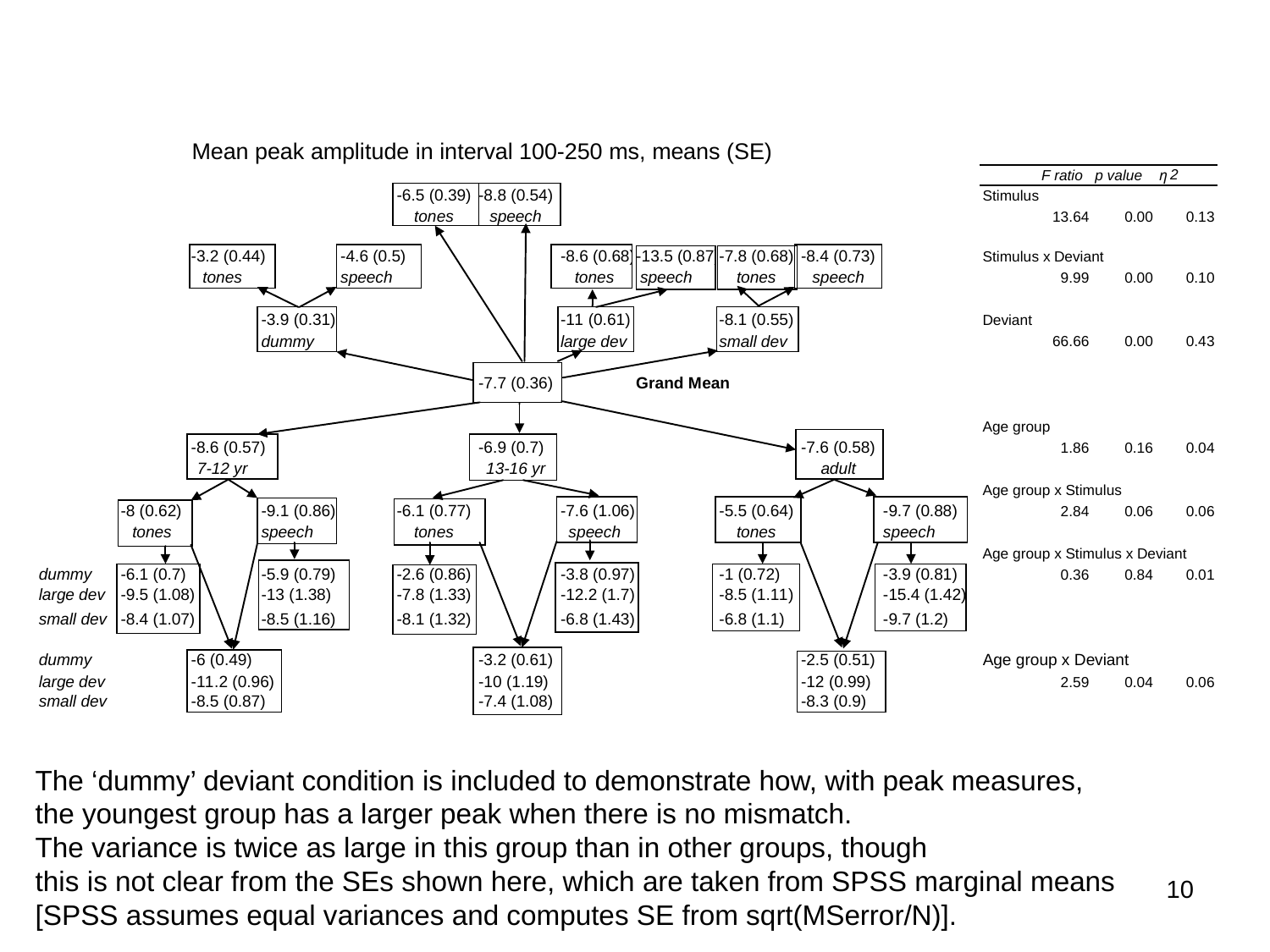

The ‘dummy’ deviant condition is included to demonstrate how, with peak measures,
the youngest group has a larger peak when there is no mismatch.
The variance is twice as large in this group than in other groups, though
this is not clear from the SEs shown here, which are taken from SPSS marginal means
[SPSS assumes equal variances and computes SE from sqrt(MSerror/N)].
<number>

## Slide 11
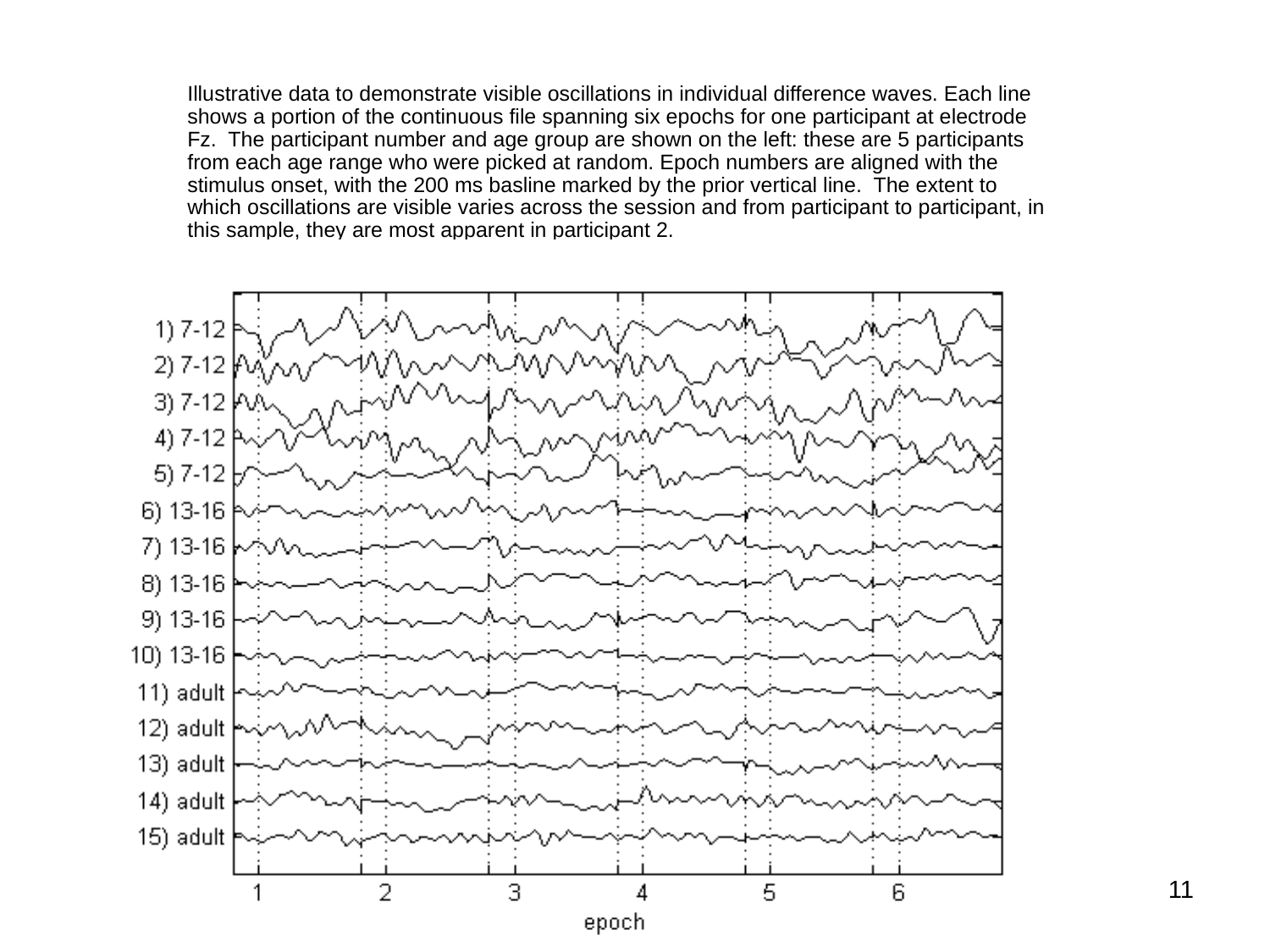

# Illustrative data to demonstrate visible oscillations in individual difference waves. Each line shows a portion of the continuous file spanning six epochs for one participant at electrode Fz. The participant number and age group are shown on the left: these are 5 participants from each age range who were picked at random. Epoch numbers are aligned with the stimulus onset, with the 200 ms basline marked by the prior vertical line. The extent to which oscillations are visible varies across the session and from participant to participant, in this sample, they are most apparent in participant 2.
<number>
